# A systematic review of colorectal multidisciplinary team meetings: an international comparison

**DOI:** 10.1093/bjsopen/zrab044

**Published:** 2021-05-20

**Authors:** M Fehervari, S Hamrang-Yousefi, M G Fadel, S C Mills, O J Warren, P P Tekkis, C Kontovounisios

**Affiliations:** Department of Surgery and Cancer, Imperial College, London, UK; Department of Colorectal Surgery, Chelsea and Westminster Hospital, London, UK; Department of Surgery and Cancer, Imperial College, London, UK; Department of Colorectal Surgery, Chelsea and Westminster Hospital, London, UK; Department of Surgery and Cancer, Imperial College, London, UK; Department of Colorectal Surgery, Chelsea and Westminster Hospital, London, UK; Department of Surgery and Cancer, Imperial College, London, UK; Department of Colorectal Surgery, Chelsea and Westminster Hospital, London, UK; Department of Surgery and Cancer, Imperial College, London, UK; Department of Colorectal Surgery, Chelsea and Westminster Hospital, London, UK; Department of Colorectal Surgery, Royal Marsden Hospital, London, UK; Department of Surgery and Cancer, Imperial College, London, UK; Department of Colorectal Surgery, Chelsea and Westminster Hospital, London, UK; Department of Colorectal Surgery, Royal Marsden Hospital, London, UK

## Abstract

**Background:**

Colorectal multidisciplinary teams (CR MDTs) were introduced to enhance the cancer care pathway and allow for early investigation and treatment of cancer. However, there are no ‘gold standards’ set for this process. The aim of this study was to review the literature systematically and provide a qualitative analysis on the principles, organization, structure and output of CR MDTs internationally.

**Methods:**

Literature on the role of CR MDTs published between January 1999 and March 2020 in the UK, USA and continental Europe was evaluated. Historical background, structure, core members, education, frequency, patient-selection criteria, quality assurance, clinical output and outcomes were extracted from data from the UK, USA and continental Europe.

**Results:**

Forty-eight studies were identified that specifically met the inclusion criteria. The majority of hospitals held CR MDTs at least fortnightly in the UK and Europe by 2002 and 2005 respectively. In the USA, monthly MDTs became a mandatory element of cancer programmes by 2013. In the UK, USA and in several European countries, the lead of the MDT meeting is a surgeon and core members include the oncologist, specialist nurse, histopathologist, radiologist and gastroenterologist. There were differences observed in patient-selection criteria, in the use of information technology, MDT databases and quality assurance internationally.

**Conclusion:**

CR MDTs are essential in improving the patient care pathway and should express clear recommendations for each patient. However, a form of quality assurance should be implemented across all MDTs.

## Introduction

The management of colorectal cancer (CRC) requires specialist knowledge and advanced decision making within a highly developed healthcare system. Effective communication and organization are important in enhancing patient outcomes and safety. One of the many parameters defining a successful healthcare system is the quality of its cancer care pathway. The outcomes of patients with CRC have improved over the last few decades due to earlier recognition and treatment improvements^1^^,^^2^, but patient management is also dependent on the organization of preoperative investigations and good teamwork. Colorectal multidisciplinary teams (CR MDTs) were introduced to be in charge of the delivery of these services.

The introduction of MDTs into cancer care has differed globally and has faced many cultural and geographical challenges. The UK was the first to introduce CR MDTs followed by the USA and continental Europe^3–8^. Each region has chosen a slightly different path and organized their MDTs following different principles and methods. The comparison of these MDTs is important in order to evaluate the best approach, facilitate learning and define the ‘gold standard’ of CR MDTs. A literature search was undertaken to investigate the principles, organization, processes and outcomes of CR MDTs and recommendations were made based on this information.

## Methods

### Search strategy

A systematic literature search was conducted concerning CR MDTs according to the protocol recommended by the Cochrane collaboration. MEDLINE and EMBASE databases were searched using the OVID platform for studies published between January 1999 and March 2020. The terms ‘colorectal cancer’ and ‘multidisciplinary team’ and their multiple synonyms were used. The exact search strategy is detailed in *Appendix S1*.

Articles were allocated into three groups (UK, USA and continental Europe) according to the geographical location they originated from, as the development of CR MDTs followed dissimilar pathways in the different regions. The search was limited to countries from regions with population over 50 million with a developed MDT system running for more than 5 years. Conference abstracts and articles in any language other than English were excluded. Initially two reviewers independently screened the titles and abstracts for inclusion. For each abstract that was not excluded, the full manuscript was read independently to determine ultimate inclusion in the final analysis. Any conflicts were resolved by a third reviewer who also confirmed that the final selected manuscripts met the inclusion criteria. A manual search of the references from selected articles and reviews which related to this research was performed to identify additional relevant studies.

### Data synthesis

Any data describing historical background, organizational or structural aspects of MDTs, education of MDT members, patient selection criteria or quality assurance (QA) were extracted. Structural differences were identified and compared between regions with a focus on identifying the most effective processes in certain regions. Risk of bias was not assessed as this study focused on the summary of available knowledge and does not directly report on treatment outcome or effect of medicine.

## Results

**Fig. 1:**
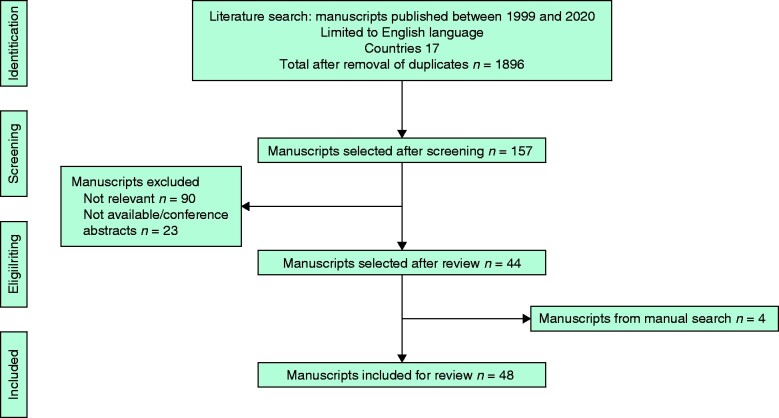
PRISMA flow diagram of studies

The literature search identified 1896 studies. All the abstracts were screened and 48 full-text articles strictly met the inclusion criteria. A preferred reporting items for systematic reviews and meta-analysis (PRISMA) flowchart of the selection process is presented in *Fig. 1*. Relevant characteristics of MDTs in the UK, Europe and the USA are presented in *Table 1*.

**Table 1:** Summary of characteristics of colorectal multidisciplinary teams worldwide

Item	UK	USA	Continental Europe
**Historical background**	Department of Health recommended cancer centres and establishment of cancer MDTs in 1995. Twenty-three cancer networks established by 2001	OSTRiCh consortium introduced CR MDTs in 2011. Canadian Institutes of Health Research established CR MDTs in 2007	First national report and recommendations for cancer centres demonstrated 91% engagement across 183 centres in 2002
**Definition**	Department of Health: group of people of different healthcare disciplines, which meet together at a given time to discuss a given patient and who are able to contribute independently to the diagnostic and treatment decisions	National Cancer Institute: treatment planning approach in which several doctors, who are experts in different specialties, review and discuss the medical condition and treatment options of a patient	Institut National du Cancer: platform to bring together the best medical cancer specialists to assist in the development of a treatment plan for patients
**Core members**	Surgeon, oncologist, specialist nurse, histopathologist, radiologist and endoscopist. MDT coordinator has been added to the list of desired core members	Surgeon, histopathologist, radiologist, specialist nurse, medical and radiation oncologist	The Netherlands and Italy: surgeon, radiologist, histopathologist, specialist nurse, radiation and medical oncologist France and Spain: geneticist, gastroenterologist, genetic counsellor, biologist, GP and psychologist
**Education**	Nationwide organized workshops complemented by mentoring events	Attending surgeons, pathologist and radiologists must complete NAPRC-endorsed education modules	Sweden: annual MDT workshop Denmark: histopathological evaluation and MRI imaging
**Frequency**	At least every fortnight	At least once a month	Italy: every fortnight France: once or twice a month
**Patient-selection criteria**	All patients with CRC discussed pre- and postoperatively	All new CRC patients as well as patients requiring further treatment due to recurrence or any other reasons including supportive/palliative treatment	Sweden: all CRC patients Italy: all rectal cancer cases Germany: after completion of primary therapy, prior to any therapy for stage IV disease
**MDT lead**	Colorectal surgeon	MDT director appointed to chair the MDT, usually the colorectal surgeon	Colorectal surgeon, however, led and coordinated by gastroenterologist in Spain and France
**Database**	Need for national database raised in 2007	MDT data stored in local databases	Germany: OncoBox electronic national database Scandinavia: colorectal cancer registry since 1993 Spain: national epidemiological registry
**Output**	Direct clinical orders followed in 85–90% of cases	Recommendations implemented in approximately 90% of cases	Referral diagnosis and stage corrected in approximately 20% of cases
**Quality assurance**	Peer-review of MDTs since 2004 (MDT-FIT self-assessment programme)	NCCCP developed a quality-assessment tool for CR MDTs	Germany, Denmark and Sweden: registry based The Netherlands: external peer review (Visitatie)
**Outcomes**	40% of advanced disease cases benefit and less favourable for patients with early disease	Reduced positive CRM in rectal cancer patients. Observed 5% survival benefit with discussion	Denmark: no perceived change Sweden: higher R0 resection rates

CRM, circumferential resection margin; CRC, colorectal cancer; CR MDT, colorectal multidisciplinary teams; GP, general practitioner; MDT, multidisciplinary team; MDT-FIT, Feedback for Improving Team-working; NAPRC, National Accredited Programme for Rectal Cancer; NCCCP, National Community Cancer Centers Program; OSTRiCh, Optimizing the Surgical Treatment of Rectal Cancer.

### Historical background of colorectal MDTs

The Department of Health in the UK was the first to recommend the development of CRC networks in 1995. The Calman–Hine report provided the background for standardizing CRC care across the whole nation^9^. The report covered mapping of the patient journey, preoperative staging, targeted preoperative strategies for chemo-radiotherapy, precision surgery, accurate pathological assessment and adjuvant therapy. Twenty-three cancer networks were established by 2001. The progress in setting up CRC networks and MDTs was evaluated a year later, with the majority of units (90 per cent) confirming the presence of CR MDT meetings held at least fortnightly^3^^,^^10^.

In Canada, the first MDTs were established in 2007 based on project Team ACCESS (Team in Access to Colorectal Cancer Services in Nova Scotia) under the supervision and funding of the Canadian Institutes of Health Research^4^. In 2011, CR MDTs were introduced in the USA with the support of OSTRiCh (Optimizing the Surgical Treatment of Rectal Cancer), a consortium with the purpose of transforming the delivery of CRC care^5^. The aim of OSTRiCh was to create Centres of Excellences (CoEs) throughout the USA with highly trained MDTs administering a care pathway despite existing health, economic and political barriers. All outcomes are recorded within 30 days and updated four times a year to a national database for QA purposes^11^. These principles are very similar to those set out in the UK. The American College of Surgeons (ACS) Commission on Cancer recognized the importance of this and monthly MDTs have been a mandatory element of cancer programmes in the USA since 2013^12–14^.

**Fig. 2:**
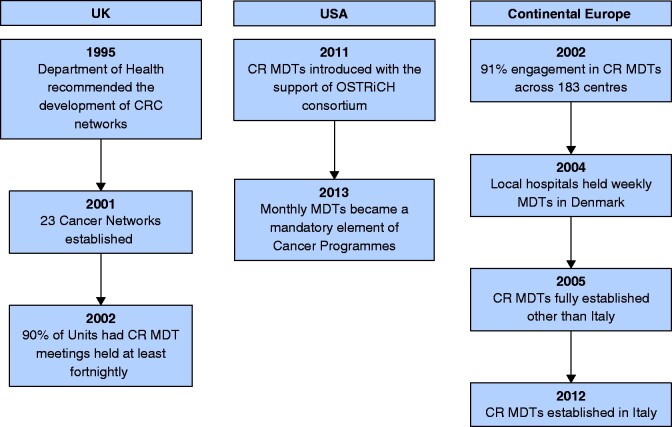
Summary of the introduction of colorectal multidisciplinary teams worldwide CR MDT, colorectal multidisciplinary team; CRC, colorectal cancer; OSTRiCh, Optimizing the Surgical Treatment of Rectal Cancer.

CR MDTs were established in Europe between 2003 and 2005. An exception is Italy, where MDTs were only fully established by 2012^6^. Similar to the UK, European MDT developments were mainly led by government bodies through National Cancer Plans and organizations such as Institut National du Cancer (France) or Oncologic National Programme (Italy)^15^. In Scandinavia, the Stockholm Colorectal Cancer Study Group has recorded outcomes prospectively for CRCs into regional registries since 1995, whilst in Copenhagen, local hospitals have held weekly MDTs since 2004^7^^,^^8^. Further development of MDTs was strongly influenced by data registration in national quality registries in the region. The introduction of CR MDTs worldwide is summarized in *Fig. 2*.

### Organizational or structural aspects of MDTs

There are no existing guidelines on who specifically should lead and attend CR MDTs. Leaders of CR MDTs should encourage participation of team members and ensure they all have a role to play^16^. Core members are frequently altered aspects of CR MDTs, being initially outlined in the Calman–Hine report and further enhanced in several studies in the UK^17^. They consist of a colorectal surgeon, oncologist, specialist nurse and gastroenterologist. Further amendments include the addition of a histopathologist, radiologist, endoscopist and geneticist. Initially, core members had to fulfil the administrative requirements. However, this reduced their time available for clinical duties resulting in an inappropriate use of limited resources^18^, and therefore an MDT coordinator has been added to the list of core members^3^. This non-clinical role is responsible for the administrative aspect, ensuring that relevant imaging and results are available for the meeting, documenting decisions and outcomes and tracking the patient’s progress. Approximately 70 per cent of UK MDTs had an appointed coordinator by 2004^10^.

Core members in the USA consist of at least one surgeon, pathologist, radiologist, medical and radiation oncologist and occasionally specialist nurses. Each member must attend at least half of all MDTs. A coordinator is appointed annually as well as a director who chairs the meeting, analyses the data submitted to the National Cancer Database (NCDB) and reports on programme performance quarterly^5^^,^^19^. In Canada, MDTs were described as an interdisciplinary team consisting of more than 20 researchers and decision makers (health services research, epidemiology, population health, primary care, psychiatry, paediatrics, and surgical, medical and radiation oncology)^4^. CR MDT meetings are commonly led by colorectal surgeons who are responsible for creating an individualized plan in the UK and North America and some parts of Europe^3^^,^^5^^,^^9^. However, in Spain it is encouraged that gastroenterologists lead the MDT, and MDTs are also often led by gastroenterologists in France^20^^,^^21^.

There is a wide range of CR MDT members in Europe. In Italy, there are four core members: surgeon, medical oncologist, radiation oncologist and radiologist. However, the MDT coordinator and nurse specialists are also desirable. Core members must attend 90 per cent of meetings, which are held at least fortnightly^6^. In Denmark, participation of local surgeons, pathologists and radiologists is encouraged in university cancer centre MDTs^6^^,^^8^^,^^22^. In the Netherlands, members are expected to be affiliated with cancer institutions^23^. In Spain, the following are considered as CR MDT members: gastroenterologist, medical and radiation oncologist, pathologist, geneticist, endoscopist, primary care physician, colorectal surgeon, specialist nurse, radiologist and clinical psychologist. The idea that the MDT should be led and coordinated by gastroenterologists is promoted^20^. Geneticists are also encouraged to attend in France^21^.

### Education of MDT members

CR MDTs are a valuable training opportunity for junior doctors by observing other specialties and senior colleagues^24^. The first organized education programme in the UK designed for MDTs was set up in 2003, which focused on rectal cancer. This workshop was complemented by mentoring events and annual workshops for specialist MDTs focusing on rectal cancer and total mesorectal excision (TME-MDT). This successful programme reached nearly all TME-MDTs in the UK by inviting 186 of them. In 2010, the programme was evaluated and the highest level of self-reported job satisfaction was reported by team members who provide direct patient care^17^.

In the USA, CR MDT meetings were initially organized as learning events providing opportunity for education rather than patient care^19^. Attending surgeons, pathologists and radiologists are mandated to complete the National Accredited Programme for Rectal Cancer (NAPRC)-endorsed education modules^5^. This ensures that evidence-based care pathways are followed by individuals undergoing parallel expert level training in their particular fields (e.g., rectal cancer MRI, pathology assessment)^11^.

In Europe, similar to the USA, MDT members have organized education since government recommendations for the establishment of MDTs were drawn up. For example, Stockholm has had an annual MDT workshop since 2004^7^. In Denmark, this is further enhanced by histopathological evaluation of the operative specimens being compared to preoperative MRIs to evaluate the accuracy of the preoperative staging. Although an educational opportunity, junior doctors have noted difficulties in attendance due to other clinical commitments^8^.

### Frequency

The frequency of MDT meetings around the world generally varies between weekly to fortnightly^6^^,^^9^^,^^19^^,^^25^^,^^26^. The exception is the USA where meetings can be held once a month, however more than two-thirds of teams hold them weekly^19^.

### Patients

In the UK, all CRCs are discussed before and after treatment. Discussion of a complex case at a specialist MDT is associated with higher survival rates^27^. This is supported by a national questionnaire suggesting that patients discussed at MDTs underwent thorough staging and assessment for non-resectable metastatic disease and fitness for surgery^28^. The benefit of MDT discussion is most marked in patients with advanced disease and clinicians are not always aware of what their colleagues in other specialties might be able to offer^29^. In the USA, as per ACS guidance, all new cancer and recurrence cases should be discussed, along with patients requiring supportive/palliative treatment^30^.

In Sweden, the focus of discussion is usually for more complex cases^7^. Indeed, in Stockholm, T1–2 disease is not always discussed and there is a focus on advanced disease. It was shown that MDT discussions resulted in more appropriate tumour staging, and R0 resections were significantly higher. In addition, this allowed for optimization of patient selection for preoperative treatment and surgery^31^.

Patients discussed in an MDT setting were more commonly selected for surgery for metastatic disease and received more targeted chemotherapy^7^. This highlights the importance of MDTs to open up the opportunity for more aggressive treatment with better outcomes. In Italy, MDT discussion is considered obligatory for all patients with rectal cancer^6^^,^^22^. In Germany, patients with metastatic disease should be re-discussed after completion of primary tumour therapy, but prior to any therapy for stage IV disease^32^.

UK data suggest that the MDT process predominantly benefits 40 per cent of patients who present with advanced disease and is less favourable for patients with early tumours. These results call into question the current belief that all new patients with CRC should be discussed at an MDT meeting^29^.

In the USA, it is also believed that tumour boards may be most beneficial for complex patient cases^33^. Patients presented at these meetings received more frequent neoadjuvant chemotherapy and their care followed the recommended standard treatment pathway^24^^,^^26^^,^^34^^,^^35^.

### Meeting structure

The CR MDT in the UK often starts with a presentation of the patient history including co-morbidities, clinical and psychological condition of the patient and pre-MDT work-up investigations. This is followed by clinical staging by each profession such as pathologist or radiologist before an agreement is reached on optimal treatment. If a decision cannot be reached, the case is forwarded to a specialist cancer institution. The average length of a meeting is 76 minutes, approximately three minutes per case^25^. The most common reasons for delays include the return of imaging or biopsy results^23^. After development of specialist CR MDTs such as rectal cancer MDT, a shift towards subspecialization amongst oncologists and radiologists has been observed. Rectal cancer MDTs led to an increase in the use of MRI for preoperative staging which requires specialist radiological knowledge^24^.

The model of CR MDT in the USA was based on the system already implemented in the UK. OSTRiCh organized rectal cancer care into CoEs throughout the USA. Evidence-based care pathways are strictly followed, reducing disparity between different regions and improving outcomes. OSTRiCh clearly outlines the principles that MDTs should follow: centres must be accredited by NAPRC, external referrals must have original pathology slides, and MDTs should be used for training, collection and data validation^11^. Despite efforts to standardize CR MDTs, there are still differences in treatment and outcomes by cancer centre type, geographical location and hospital volume. Nevertheless, adherence to guidelines is being reviewed by external validation processes^36^. A large survey of US institutions emphasized the importance of a moderator and specific criteria for selecting cases, and written summaries before meetings to improve time management^19^.

In Italy, there is a focus on improving utilization of the MDT. The clinical history, imaging and histopathology are reviewed and all data are captured for audit and monitoring purposes. Case notes, diagnostic data, staging and pathological information must be available^22^. The availability of up-to-date resources and information on patient preferences aids clinical decision making on conservative *versus* surgical management. In Denmark, conferences are often arranged locally and there is a variability in structure, although guidelines on their organization have been provided^8^. In Sweden, radiological imaging, in particular MRI scans, is discussed primarily, leading to detailed staging and targeted treatment^37^.

### Clinical output and outcome of CR MDTs

The most common outputs of MDTs are direct clinical orders, followed in approximately 90 per cent of cases in the UK^36^^,^^38^. Lack of compliance with recommendations is related to patient co-morbidities and patient preferences^36^. The National Cancer Action Team (NCAT) published the Characteristics of an Effective MDT report, which defined the clinical document a CR MDT should produce^39^.

In the USA, MDTs signiﬁcantly inﬂuence preoperative decision making, such as choice of staging modality and neoadjuvant treatment. A survey of MDT members found that tumour board recommendations were generally implemented in at least 90 per cent of cases^36^. The most common cause of diversion from MDT decision was patient choice. Regular MDT meetings improved guideline adherence and quality of rectal cancer care^34^ and increased clinical trial participation^26^.

A postoperative audit of positive circumferential resection margins (CRMs) suggests MDT discussions result in significantly reduced positive CRM in rectal cancer patients. Only 8 per cent of patients having MDT discussion of MRI had positive margins compared with the national average of 20 per cent^28^.

MDTs in Europe are reported to rectify the referral diagnosis and clinical stage in 22 per cent of evaluated patients^40^. The presence of treating physicians is the most inﬂuential variable to ensure a correct diagnosis and stage. Most of the altered diagnoses formulated after re-review of imaging and pathology, or new ﬁndings, result from additional diagnostic tests. The need for highly specialized care should be highlighted during local CR MDT meetings to ensure patients receive appropriate and timely treatment^41^.

In Denmark, there was an improvement in postoperative mortality after the introduction of MDT conferences. However, there was no change in local recurrence and overall survival, despite more precise preoperative staging and an increase in MRI imaging performed in the MDT cohort^8^.

### Databases

MDT registries are databases where clinical data are logged prospectively at a local, regional or national level. The UK was the first to develop automatic mandatory recording of data to a uniform national database. In 2007, it was highlighted that such a database would facilitate communication between MDTs within networks, and also underpin a national audit via NCDB^18^.

In the USA, similar to the UK, MDT data are stored in local databases, which are then reviewed externally for QA^36^. There are no data suggesting there are prospective national CRC databases across the USA.

Databases are more uniform nationwide in Europe. In Germany, all data from the tumour documentation of respective centres are submitted electronically to a software, OncoBox, that can interact with and extract data from the tumour-documentation software used in the entire country. This runs a plausibility data check and calculates the quality indicators, and discrepancies are fed back to centres for clearance^33^. The Norwegian Colorectal Cancer Registry, established in 1993, has become part of the National Cancer Registry^42^. In Sweden, the Stockholm Colorectal Cancer Study Group was set up in 1980 to improve CRC outcomes^7^. In Spain, a national epidemiological registry for high-risk patients is promoted, including genetic counselling training for medical oncologists and risk-management protocols^20^.

### Quality assurance

The most common form of QA is external peer review, which can improve quality of care through more rigorous adherence to best practice guidance^43^. There is an existing standard for MDT reports in the UK, which provides a working deﬁnition for the assessment and development of MDTs. Based on adherence to the standards, NCAT has developed an MDT self-assessment programme known as MDT–FIT (Feedback for Improving Team-working), which is aimed at supporting teams to self-evaluate their performance and receive feedback^38^. Peer review of CR MDTs was also introduced in 2004^43^.

In the USA, the National Cancer Institute Community Cancer Centres Program developed an assessment tool based on a consensus by the participating units to evaluate the quality of CR MDTs^44^.

In Europe, external QA programmes of CR MDTs are increasingly considered to be essential in the improvement of quality of CR MDTs. In the Netherlands, external peer review is the dominant external QA method. This programme targets the multidisciplinary cancer care organization in hospitals as a whole, whilst in the UK programmes focus on individual tumour groups^43^. In Germany, the National Cancer Programme aims to ensure evidence-based guideline recommendations and MDTs are heavily involved in cancer care.

### MDT assessment

There are numerous attempts to capture the positive influence of MDTs on patient outcomes. However, it is difficult to assess for various reasons, such as heterogeneity of data, difference in care standards and limitations of study designs^16^^,^^45^.

MDT presentation and reporting were investigated using The Colorectal Multidisciplinary Team Metric for Observation of Decision-Making (cMDT-MODe)^25^ in the UK and with the Multidisciplinary Clinics and Conferences Assessment Tool (MDC) in the USA^44^.

A study reported how MDTs also facilitate collaboration between surgeons and oncologists. For each patient shared per year between specialists, there was an observed survival benefit from colon cancer-specific mortalities^46^.

## Discussion

An MDT approach in the treatment of CRC is essential to patient care. The purpose of this review was to describe how CR MDTs are organized and structured across different countries internationally. Regional comparison revealed significant variation in the MDT process, however there are differences between meetings within each region, leading to differences in treatment options and outcome^16^.

The most common issues with MDTs described are a lack of staffing and resources. MDT meetings may not be part of a clinician’s job plan and often clash with other activities resulting in non-attendance. Apart from a clinical panel, MDTs require significant administrative and technical support in the form of at least one MDT clerk. There is still a lack of non-clinical support globally which manifests in a loss of clinician time and a lack of documentation^16^. It is clear that all MDT participants should have allocated administrative time in their job plan for MDTs. MDT meetings are most commonly led by surgeons, however gastroenterologists are found to be more appropriate leaders in some institutions.

The criteria for patients to be discussed traditionally were patients with a possible diagnosis of cancer. The costs of MDT meetings can be fairly high and some areas, such as Scandinavia, felt that discussing localized disease in MDTs produced little clinical value^6^^,^^47^^,^^48^. On the other hand, some nations, such as the UK, believe that the stage of the disease is not the only important factor influencing treatment and all stages should be discussed. Changes in outcome following altered patient selection should be monitored very closely. Appropriate changes may improve the use of valuable resources, however MDTs must make sure that treatment outcomes are not affected. Legal issues and internal QA are other important factors in patient selection for CR MDT.

The most common reason for MDT recommendations not being implemented or being postponed are no record of patient’s choice and co-morbidities and lack of investigation results. Electronic patient records and electronic referral forms have been established in the UK for several years, however many MDT referrals are still being received by electronic and postal mail. A development of a comprehensive colorectal MDT checklist could significantly improve the efficiency and adherence to recommendations of colorectal and other MDTs. This checklist should include information regarding fitness for surgery, patient choice of treatment, which should not change the optimal MDT recommendation, but should prompt the MDT to discuss alternative treatment strategies simultaneously. The checklist should be kept updated with final treatment decision, and operative and histological outcome to ensure optimal information flow throughout the patient journey.

Databases have already been established in all examined countries, however, in the UK and USA these are predominantly local databases. In continental Europe, there are excellent examples, such as Germany, of how national databases can simultaneously support clinical activity and research. Education of MDT members is clearly improving job satisfaction, ensuring a smoother patient flow and stricter adherence to guidelines. Some countries, such as the USA, are ahead with educating MDTs due to historical reasons.

Currently the most common form of QA of MDTs are external peer reviews. This is a widely accepted method; however, these reviews are often subjective. There are a few examples of automatic software-based QA. These methods are less biased, provide regular feedback at a team level and are more efficient than external reviews, hence they should be implemented across all MDTs.

Regarding the limitations of this study, different healthcare systems tailored colorectal multidisciplinary discussion meetings to their existing decision-making pathways. This led to minor structural differences between meetings which make comparison difficult. The available studies often described different parameters of the CR MDT process which provide challenges in the synthesis of cumulative data. Furthermore, there are no, and likely will never be, randomized studies comparing patients undergoing or not undergoing MDT discussion. There also aspects of team dynamics, such as difference in leadership or effect of hierarchy, that are difficult to capture.

The most common problems CR MDTs encounter are related to funding, staffing and delays in decision making due to inaccessible results. The latter could potentially be improved by implementing pre-MDT checklists. Some recent evidence suggests limited benefit of MDTs for patients with early disease. The UK was the first to introduce CR MDT meetings, education of MDT members and organized cancer networks. In the USA, MDT meetings were started as an education event for junior doctors and aspects of patient care were later introduced. These meetings remain highly rated educational events, which should be the case for all MDTs. In continental Europe, major advances have been made in the development of national databases which link cancer centres together, and this provides an excellent foundation for automatized QA, which all CR MDTs should have in place.

## Supplementary Material

zrab044_Supplementary_Data
