# Supplementary material for: A systematic review of colorectal multidisciplinary team meetings: an international comparison
Source: BJS Open. 2021 May 20;5(3):zrab044. doi: 10.1093/bjsopen/zrab044 (PMC8134530; doi:10.1093/bjsopen/zrab044)
Supplement: zrab044_Supplementary_Data [file zrab044_supplementary_data.zip › Appendix 1.docx]

| **#** | **Database** | **Search term** | **Results** |
| --- | --- | --- | --- |
| 1 | Medline | (colon* cancer OR colon* neoplasm* OR colon* malignan* OR colon* carcinoma* OR colon* adenocarcinoma* OR colon* tumo?r).ti,ab | 138863 |
| 2 | Medline | (rect* cancer OR rect* neoplasm* OR rect* malignan* OR rect* carcinoma* OR rect* adenocarcinoma* OR rect* tumo?r).ti,ab | 53633 |
| 3 | Medline | (bowel cancer OR bowel neoplasm* OR bowel malignan* OR bowel carcinoma* OR bowel adenocarcinoma* OR bowel tumo?r).ti,ab | 30058 |
| 4 | Medline | (sigmoid cancer OR sigmoid neoplasm* OR sigmoid malignan* OR sigmoid carcinoma* OR sigmoid adenocarcinoma* OR sigmoid tumo?r).ti,ab | 5921 |
| 5 | Medline | (colorectal cancer OR colorectal neoplasm* OR colorectal malignan* OR colorectal carcinoma* OR colorectal adenocarcinoma* OR colorectal tumo?r).ti,ab | 117158 |
| 6 | Medline | (cancer OR neoplasm* OR malignan* OR carcinoma* OR adenocarcinoma* OR tumo?r).ti,ab | 2802559 |
| 7 | Medline | (colon* OR rect* OR bowel OR sigmoid OR colorectal).ti,ab | 687111 |
| 8 | Medline | (6 AND 7) | 279634 |
| 9 | Medline | "COLORECTAL NEOPLASMS"/ OR "COLONIC NEOPLASMS"/ OR "COLORECTAL NEOPLASMS, HEREDITARY NONPOLYPOSIS"/ OR "RECTAL NEOPLASMS"/ OR "SIGMOID NEOPLASMS"/ | 178028 |
| 10 | Medline | (1 OR 2 OR 3 OR 4 OR 5 OR 8 OR 9) | 317720 |
| 11 | Medline | (multidisciplinary OR multi-disciplinary OR interdisciplinary).ti,ab | 106894 |
| 12 | Medline | (team* OR meeting* OR communication* OR approach* OR care OR treatment*).ti,ab | 6406308 |
| 13 | Medline | (11 AND 12) | 86745 |
| 14 | Medline | (multidisciplinary team* OR multi-disciplinary team* OR multidisciplinary meeting* OR multi-disciplinary meeting* OR multidisciplinary communication* OR multi-disciplinary communication* OR multidisciplinary approach* OR multi-disciplinary approach* OR multidisciplinary care OR multi-disciplinary care OR multidisciplinary treatment* OR multi-disciplinary treatment*).ti,ab | 64179 |
| 15 | Medline | (interdisciplinary team* OR interdisciplinary meeting* OR interdisciplinary communication* OR interdisciplinary approach* OR interdisciplinary care OR interdisciplinary treatment*).ti,ab | 22211 |
| 16 | Medline | (decision making OR clinical decision making OR patient care team OR oncology team OR oncoteam OR tumo?r board*).ti,ab | 141028 |
| 17 | Medline | (13 OR 14 OR 15 OR 16) | 218247 |
| 18 | Medline | (10 AND 17) | 4250 |
| 19 | Medline | 18 [DT 1999-2019] [Languages English] | 3523 |
| 20 | Medline | "PATIENT CARE TEAM"/ | 61962 |
| 21 | Medline | (17 OR 20) | 264292 |
| 22 | Medline | (10 AND 21) | 4457 |
| 23 | Medline | 22 [DT 2014-2019] [Languages English] | 1799 |
| 24 | EMBASE | (colon* cancer OR colon* neoplasm* OR colon* malignan* OR colon* carcinoma* OR colon* adenocarcinoma* OR colon* tumo?r).ti,ab | 84382 |
| 25 | EMBASE | (rect* cancer OR rect* neoplasm* OR rect* malignan* OR rect* carcinoma* OR rect* adenocarcinoma* OR rect* tumo?r).ti,ab | 38203 |
| 26 | EMBASE | (bowel cancer OR bowel neoplasm* OR bowel malignan* OR bowel carcinoma* OR bowel adenocarcinoma* OR bowel tumo?r).ti,ab | 4813 |
| 27 | EMBASE | (sigmoid cancer OR sigmoid neoplasm* OR sigmoid malignan* OR sigmoid carcinoma* OR sigmoid adenocarcinoma* OR sigmoid tumo?r).ti,ab | 754 |
| 28 | EMBASE | (colorectal cancer OR colorectal neoplasm* OR colorectal malignan* OR colorectal carcinoma* OR colorectal adenocarcinoma* OR colorectal tumo?r).ti,ab | 148444 |
| 29 | EMBASE | (cancer OR neoplasm* OR malignan* OR carcinoma* OR adenocarcinoma* OR tumo?r).ti,ab | 3682495 |
| 30 | EMBASE | (colon* OR rect* OR bowel OR sigmoid OR colorectal).ti,ab | 1004807 |
| 31 | EMBASE | (29 AND 30) | 413099 |
| 32 | EMBASE | "COLON CANCER"/ OR "RECTUM CANCER"/ OR "COLORECTAL CANCER"/ OR "COLORECTAL CARCINOMA"/ OR "METASTATIC COLORECTAL CANCER"/ OR "COLON CARCINOMA"/ OR "SIGMOID CANCER"/ OR "METASTATIC COLON CANCER"/ OR "RECTUM CARCINOMA"/ | 258204 |
| 33 | EMBASE | (24 OR 25 OR 26 OR 27 OR 28 OR 31 OR 32) | 467778 |
| 34 | EMBASE | (multidisciplinary OR multi-disciplinary OR interdisciplinary).ti,ab | 167920 |
| 35 | EMBASE | (team* OR meeting* OR communication* OR approach* OR care OR treatment*).ti,ab | 8344270 |
| 36 | EMBASE | (34 AND 35) | 140371 |
| 37 | EMBASE | (multidisciplinary team* OR multi-disciplinary team* OR multidisciplinary meeting* OR multi-disciplinary meeting* OR multidisciplinary communication* OR multi-disciplinary communication* OR multidisciplinary approach* OR multi-disciplinary approach* OR multidisciplinary care OR multi-disciplinary care OR multidisciplinary treatment* OR multi-disciplinary treatment*).ti,ab | 62307 |
| 38 | EMBASE | (interdisciplinary team* OR interdisciplinary meeting* OR interdisciplinary communication* OR interdisciplinary approach* OR interdisciplinary care OR interdisciplinary treatment*).ti,ab | 13881 |
| 39 | EMBASE | (decision making OR clinical decision making OR patient care team OR oncology team OR oncoteam OR tumo?r board*).ti,ab | 154922 |
| 40 | EMBASE | "MULTIDISCIPLINARY TEAM"/ OR "COLLABORATIVE CARE TEAM"/ | 1850 |
| 41 | EMBASE | (36 OR 37 OR 38 OR 39 OR 40) | 289271 |
| 42 | EMBASE | (33 AND 41) | 7625 |
| 43 | EMBASE | 42 [DT 2014-2019] [English language] [Languages English] | 3612 |
| 44 | Medline | (united kingdom OR UK OR england OR scotland OR wales OR northern ireland OR great britain OR ireland OR republic of ireland OR france OR switzerland OR denmark OR finland OR norway OR austria OR belgium OR germany OR netherlands OR holland OR sweden OR spain OR italy OR greece).ti,ab | 589922 |
| 45 | Medline | GERMANY/ OR NETHERLANDS/ OR SPAIN/ OR ITALY/ OR GREECE/ OR FRANCE/ OR IRELAND/ OR SWITZERLAND/ OR BELGIUM/ OR AUSTRIA/ OR exp "UNITED KINGDOM"/ OR DENMARK/ OR FINLAND/ OR NORWAY/ OR SWEDEN/ | 1013494 |
| 46 | Medline | (44 OR 45) | 1278185 |
| 47 | Medline | (north america OR united states of America OR usa OR united states OR canada OR mexico).ti,ab | 418049 |
| 48 | Medline | "NORTH AMERICA"/ OR CANADA/ OR MEXICO/ OR "UNITED STATES"/ | 965703 |
| 49 | Medline | (47 OR 48) | 1223154 |
| 50 | Medline | (18 AND 46) | 502 |
| 51 | Medline | (22 AND 49) | 340 |
| 52 | Medline | 50 [DT 1999-2019] [Languages English] | 413 |
| 53 | Medline | 51 [DT 1999-2019] [Languages English] | 305 |
| 54 | EMBASE | (united kingdom OR UK OR england OR scotland OR wales OR northern ireland OR great britain OR ireland OR republic of ireland OR france OR switzerland OR denmark OR finland OR norway OR austria OR belgium OR germany OR netherlands OR holland OR sweden OR spain OR italy OR greece).ti,ab | 999916 |
| 55 | EMBASE | GERMANY/ OR NETHERLANDS/ OR GREECE/ OR ITALY/ OR SPAIN/ OR AUSTRIA/ OR BELGIUM/ OR FRANCE/ OR IRELAND/ OR SWITZERLAND/ OR DENMARK/ OR FINLAND/ OR NORWAY/ OR SWEDEN/ OR exp "UNITED KINGDOM"/ | 1123534 |
| 56 | EMBASE | (54 OR 55) | 1579345 |
| 57 | EMBASE | (north america OR united states of America OR usa OR united states OR canada OR mexico).ti,ab | 598869 |
| 58 | EMBASE | exp "NORTH AMERICA"/ | 1347773 |
| 59 | EMBASE | (57 OR 58) | 1643399 |
| 60 | EMBASE | (42 AND 56) | 940 |
| 61 | EMBASE | (42 AND 59) | 569 |
| 62 | EMBASE | 60 [DT 1999-2019] [English language] [Languages English] | 863 |
| 63 | EMBASE | 61 [DT 1999-2019] [English language] [Languages English] | 540 |
| 64 | Medline | 50 [DT 2019-2020] [Languages English] | 76 |
| 65 | Medline | 51 [DT 2019-2020] [Languages English] | 44 |
| 66 | EMBASE | 60 [DT 2019-2020] [English language] [Languages English] | 132 |
| 67 | EMBASE | 61 [DT 2019-2020] [English language] [Languages English] | 82 |

Appendix 1. Full search strategy
